# Beyond the classroom lecture: Liang Wang’s personal war on tuberculosis in China

**DOI:** 10.1007/s13238-016-0304-3

**Published:** 2016-08-02

**Authors:** Ming Li, Xiaomei Hu, Fuquan Hu, Xiancai Rao

**Affiliations:** Department of Microbiology, College of Basic Medical Sciences, Third Military Medical University, Chongqing, 400038 China

Tuberculosis, historically one of the deadliest and most prevalent infectious diseases, is caused by the bacterium *Mycobacterium tuberculosis*. It is estimated that one-third of the world’s population is infected by this agent (Zumla et al., 2014), and tuberculosis is therefore a major topic for medical students. *M. tuberculosis* is a small aerobic bacillus, with a remarkably high lipid-content cell wall that plays a critical role in its pathogenicity. Although the efficacy of the Bacillus Calmette-Guérin (BCG) vaccine for preventing tuberculosis continues to be debated (Villarreal-Ramos, 2009), live attenuated BCG has been the only approved vaccine against *M. tuberculosis* infection since its introduction in 1921, and is still administered routinely around the world, particularly in developing countries. In China, BCG has been included in the national vaccination program for newborns and children under 15 since the mid-1970s. After decades of intensive effort, remarkable progress has been made in the fight against tuberculosis in China. This great achievement was made possible by Liang Wang, a physician who pioneered the introduction of the BCG vaccine to China in the early 1930s and spearheaded China’s fight against tuberculosis (Yan, 2003; Dai et al., 2014).

Although his accomplishments are frequently discussed in the medical school classroom, most students are unfamiliar with Liang Wang’s personal story and the significant challenges he faced in his battle against tuberculosis. Liang Wang was born on May 5, 1891 in Chengdu in the Sichuan Province. He lost his father at very young age. After graduating from the Hanoi Medical School in Vietnam in 1913, he entered private practice in the Yunnan and Sichuan regions of China. In the early 20th century, tuberculosis was the leading cause of death. Because there was no known cure, the severe health threat posed by tuberculosis was considered to be as serious as that posed by cancer today. Wang’s older brother and younger sister both died of tuberculosis. As a young doctor, Liang Wang determined to devote himself completely to the fight against tuberculosis.

In 1924, Albert Calmette and Camille Guérin from the Pasteur Institute in France published their research results on the BCG vaccine, demonstrating its efficacy in the prevention of tuberculosis. One year later, Liang Wang learned about BCG and began fundraising to support a trip to France. With the help of France’s foreign ministry, Wang was able to visit the Pasteur Institute in 1931, where he began his research career under the personal guidance of Guérin. Wang’s modesty and eagerness to learn were quickly appreciated. During his two years at the Pasteur, Wang completed four research papers, three of which focused on BCG and the culture of *M. tuberculosis*. In the summer 1933, Liang Wang returned to China with the BCG seed strain and lab equipment. He established a microbiology laboratory while working as a physician in a private hospital in Chongqing. He committed much of his spare time to BCG culture preparation and developed the first batch of BCG vaccine in China. Beginning October 1933 through August 1935, 248 infants were inoculated with the BCG vaccine manufactured by Liang Wang. No adverse reactions were observed. Crucially, BCG vaccination conferred significant protection against the development of tuberculosis, and also offered some degree of immunity against other common infectious diseases.

However, just as Liang Wang was planning to expand and promote BCG vaccination, the Japanese invaded China in 1937, beginning the Anti-Japanese War, also known as the Second Sino-Japanese War. Wang’s lab was forced to close, and the development of the vaccination project halted for more than a decade until the Japanese were defeated and the People’s Republic of China was established.

**Figure 1:**
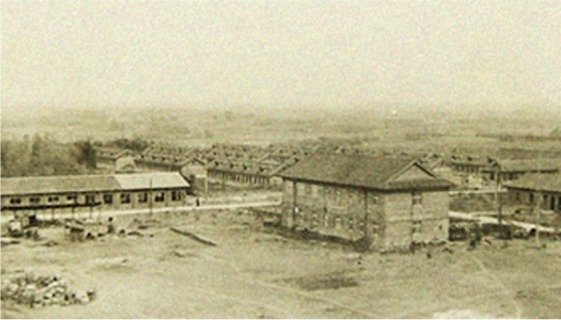
The Chengdu Institute of Biological Products in its early days

The new Department of Health, opened by the Southwest Military Administrative Committee in Chongqing, provided Liang Wang with the opportunity continuing his work. He was invited to participate in the first national health working conference in 1950, and was authorized by the central government to found the Southwest Chinese BCG Production and Research Institute in Chongqing under his leadership. Beginning August 1951, the health ministry held three training classes to promote the use of Wang’s BCG vaccine, and BCG vaccination quickly became popularized. In 1956, the Southwest BCG Production and Research Institute was incorporated into the Chengdu Institute of Biological Products (Fig. 1), where Liang Wang devoted the later stages of his professional career to BCG research and manufacturing.

For the next 30 years, Wang insisted on the priority of clinical application and the principle of combining theory with practice. By focusing on three specific areas of research, he contributed significantly to the refinement of BCG-based immunization strategies to control tuberculosis in China.

First, Wang emphasized the improvement of BCG vaccine quality. Wang optimized the chemical composition of BCG by decreasing the content of monosodium glutamate from 8% to 6% and glycerol from 6% to 5%, making it more suitable for live attenuated BCG. He also determined the most effective concentration of sucrose (8%) for the protection of lyophilized BCG preparations by using the optimum seeking method. To determine the shelf life for the vaccine, Wang measured the loss in potency for 10 different batches of BCG vaccine after storage at 4°C for 6 weeks, and confirmed that 26% of bacteria remained viable, a level sufficient to induce the body’s immune response.

Second, Wang began a process to isolate a superior BCG strain for vaccine production. Due to different passage methods and culture conditions, the BCG vaccine used in different countries varied. In collaboration with others, both locally and nationally, Liang Wang performed a two-year-long BCG breeding program in which he compared a large number of domestic and international BCG strains and established efficient detection protocols. His achievements provided the experimental basis for the selection of the Shanghai D2 strain from Denmark as the foundation for BCG vaccine production.

Third, Wang actively investigated the immunologic mechanisms underlying BCG efficacy. In an analysis of various blood constituents in BCG-immunized animals, he observed that the number of leukocytes increased significantly 3 days after vaccination, peaked on day 5, and returned to normal on day 12. Lymphocytes were the most markedly increased cell type. Through many experiments, he determined that the cytophagic capacity of reticuloendothelial cells and phagocytes were remarkably enhanced in BCG-vaccinated animals, and serum immunoglobulins were also significantly elevated. Wang also noted that the protection conferred by BCG vaccination persists even after allergies have disappeared in immunized animals. His work demonstrated that BCG induces not only cell-mediated immunity but also humoral immunity, consistent with the non-specific immunity observed in infants enrolled in Wang’s first vaccination project in 1935. Furthermore, Wang also found that BCG-immunized animals exhibited an enhanced ability to resist infection caused by *Staphylococcus aureus* and other species from the genus *Salmonella* and *Streptococcus*. This remarkable discovery provided the foundation for the clinical application of BCG and its active ingredients as immunological treatments for cancer and other diseases, and defined the future direction for development of a series of therapeutic BCG-associated products.

With his strong work ethic and rigorous attitude toward research, Liang Wang devoted himself wholeheartedly to his anti-tuberculosis career. He was invariably courteous, amiable, and easy to approach, and paid close attention to the cultivation of young scientists and physicians. He remained active as a writer and lecturer even when nearly 90 years old. In 1983, an academic symposium was organized by the Chengdu Institute of Biological Products to celebrate the 50th anniversary of Wang’s professional career and his 92th birthday.

On August 31, 1985, Liang Wang died at the age of 94 in his hometown of Chengdu.
